# Tonsillar CD56brightNKG2A+ NK cells restrict primary Epstein-Barr virus infection in B cells via IFN-γ

**DOI:** 10.18632/oncotarget.14045

**Published:** 2016-12-20

**Authors:** Aurelia Jud, Monika Kotur, Christoph Berger, Claudine Gysin, David Nadal, Anna Lünemann

**Affiliations:** ^1^ Children's Research Center, University Children's Hospital, Experimental Infectious Diseases and Cancer Research, Zurich, Switzerland; ^2^ Division of Infectious Diseases, and Children's Research Center, University Children's Hospital Zurich, Zurich, Switzerland; ^3^ Children's Research Center, University Children's Hospital, ENT Clinic, Zurich, Switzerland

**Keywords:** natural killer cells, epstein barr virus, interferon gamma, secondary lymphoid organs, B cells

## Abstract

Natural killer (NK) cells constitute the first line of defense against viruses and cancers cells. Epstein–Barr virus (EBV) was the first human virus to be directly implicated in carcinogenesis, and EBV infection is associated with a broad spectrum of B cell lymphomas. How NK cells restrict EBV-associated oncogenesis is not understood. Here, we investigated the efficacies and mechanisms of distinct NK cell subsets from tonsils, the portal of entry of EBV, in limiting EBV infection in naïve, germinal center-associated and memory B cells. We found that CD56^bright^ and NKG2A expression sufficiently characterizes the potent anti-EBV capacity of tonsillar NK cells. We observed restriction of EBV infection in B cells as early as 18 hours after infection. The restriction was most efficient in naïve B cells and germinal center-associated B cells, the B cell subsets that exhibited highest susceptibility to EBV infection *in vitro*. IFN-γ release by and partially NKp44 engagement of CD56^bright^ and NKG2A positive NK cells mediated the restriction that eventually inhibited B-cell transformation. Thus, harnessing CD56^bright^NKG2A^+^ NK cell function might be promising to improve treatment strategies that target EBV-associated B cell lymphomas.

## INTRODUCTION

Epstein Barr virus (EBV) has been strongly associated with B-cell cancers including endemic Burkitt's lymphoma, Hodgkin's disease, and post-transplant lymphoproliferative disease (PTLD) [1–3]. Immunocompromised hosts are at highest risk for EBV-associated B cell malignancies whereas the vast majority of immunocompetent individuals do not develop such pathology and remain healthy EBV carriers after primary infection, which is mainly acquired in childhood (2). Nevertheless, after Infectious Mononucleosis, i.e. symptomatic primary EBV infection, there is a 20-fold increased risk to develop Hodgkin lymphoma [4]. Of further note, EBV-associated Burkitt's lymphoma, Hodgkin's lymphoma as well as PTLD all derive from germinal center cells and are thereby linked to distinct differentiation stages of B cells in secondary lymphoid organs [5]. While the lack of EBV-specific immune control is a well-recognized risk factor for EBV-associated B cell tumors (2, 3), the factors that prevent the development of EBV-associated malignancies or allow malignant transformation in immunocompetent EBV carriers remain incompletely understood, especially those of innate immunity.

Innate immune reactions and their balanced surveillance build the first line of defense against infectious agents and cancer cells. If this balance is skewed, severe infections or neoplasms may develop. Natural killer (NK) cells were first described and named based on their innate ability to kill virus-infected cells or cancer cells without pre-activation [6, 7]. In the peripheral blood, two main NK cell subsets have been described (reviewed in [8]). The CD56^dim^ NK cells are innate cytotoxic killer cells, whereas CD56^bright^ cells produce an array of cytokines such as IFN-γ, TNF-α, GM-CSF. After their precursor development in the bone marrow NK cells migrate to secondary lymphoid organs (SLOs) including tonsils, the portal of entry of EBV, to mature and only after maturing fully egress back to the peripheral blood to patrol the human body against intruders or malignantly altered cells [9, 10]. Notably, CD56^bright^ NK cells are the most advanced differentiation stage of mature NK cells in the tonsils, whereas they are the least advanced differentiation stage of mature NK cells circulating in the peripheral blood [11]. It is long documented that NK cells are a crucial component of immune control of EBV infection and tonsillar CD56^bright^ NK cells have been found to restrict EBV-induced transformation more potently than tonsillar CD56^dim^ NK cells [12]. We have recently identified a distinct subset of tonsillar and blood CD56^bright^NKG2A^+^CD94^+^CD54^+^CD62L^−^ NK cells, which mediates restriction of EBV-induced B cell transformation after established EBV-infection [13]. Subsequently, NK cells were shown to control EBV infection in a “humanized” mouse model reconstituted with a human immune system. In this model a subset of NK cells, namely NKG2A^+^ NK cells accumulated in the peripheral blood 4 weeks after primary EBV infection, before the onset of infectious mononucleosis (IM, acute symptomatic EBV infection) like symptoms, preceding the IM characteristic CD8^+^ T cell expansion. If NK cells were depleted the experimental animals got sick more rapidly and more severely, with worse IM symptoms and a significantly higher tumor load [14]. In addition, we found recently that CD56^dim^NKG2A^+^ NK cells accumulate in the peripheral blood of acutely EBV-infected pediatric patients manifesting IM [15]. Even 6 months after IM expansion of this specific NK cell subset prevailed that preferentially targets B cells with replicating EBV.

Here, we expanded our previous work (13, 17) by investigating the efficacies and mechanisms of human tonsillar NK cell subsets to restrict early EBV infection. To this end, we compared and characterized the potency of tonsillar CD56^bright^NKG2A^+^ NK cells vs. other NK cell subsets (i) in restricting EBV-induced B cell transformation *in vitro*; (ii) in restriction of early EBV infection in B cells; and characterized (iii) the CD56^bright^NKG2A^+^ NK cells, i.e. the distinct anti-EBV NK cell subset in detail towards sorted autologous tonsillar B cell subsets, i.e. naïve, germinal center-associated, and memory B cells, respectively.

## RESULTS

### Tonsillar CD56^bright^ NKG2A^+^ NK cells restrict outgrowth of EBV-infected B cells more efficaciously than CD56^bright^ NKG2A^−^ and CD56^dim^ NK cells

We previously demonstrated that human tonsillar CD56^bright^NKG2A^+^CD94^+^CD54^+^CD62L^−^ NK cells, which produce high levels of IFN-γ, potently restrict EBV-transformed B cells *in vitro* [13]. After recent descriptions of accumulation of NKG2A^+^ NK cells during acute primary EBV infection in a humanized mouse model, with increased tumor rates after NK depletion [14] and in peripheral blood of IM patients [15], we were intrigued to determine the EBV restriction capacity of tonsillar CD56^bright^NKG2A^+^ compared to other tonsillar NK cell subsets in our previously described transformation restriction assay [13].

**Figure 1:**
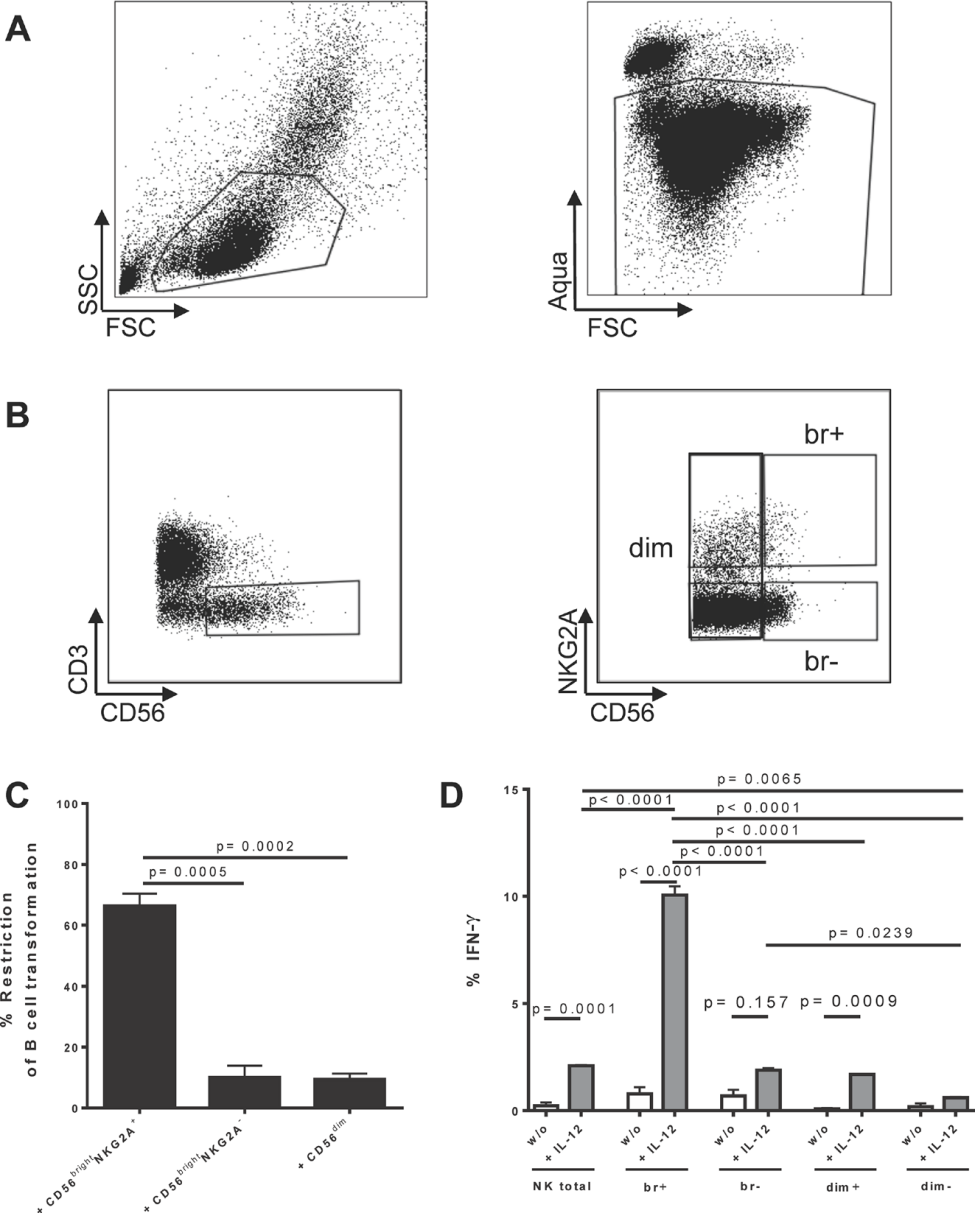
Identification and characterization of human CD56brightNKG2A+ NK cells and restriction of EBV in B cells (**A**) Basic gating for all tonsillar and peripheral samples throughout this manuscript (representative example of PBMCs is shown); (**B**) Gating for CD56^bright^NKG2A^+^ and compared NK cell subsets (representative example of PBMCs is shown); (**C**) Restriction of EBV transformation of B cells after established EBV infection in B cells by tonsillar CD56^bright^NKG2A^+^ NK cells compared to other subsets (6 donors); (**D**) IFNγ production induced by overnight cytokine stimulation in different tonsillar NK subsets (3 donors); Restriction = (100 − (% transformed B cells in co-culture/% transformed B cells without NK) ×100); Data represent three independent experiments. All error bars represent SEM. *p* values depicted were calculated with two-tailed student's *t* test, or (1D) regular ANOVA corrected for multiple comparison testing by Tukey correction.

Thus, we first determined the potency of NK cells defined by CD56 and NKG2A expression (Gating/sorting strategy Figure 1A and 1B) to restrict transformed B cells after established EBV infection as described previously [13]. Briefly, we sorted tonsillar CD56^bright^NKG2A^+^ NK cells, CD56^bright^NKG2A^−^ NK cells, and CD56^dim^ NK cells and co-cultured these subsets with autologous B cells. We quantified restriction of B cell transformation normalized to frequencies of infected B cells in cultures without NK cells with the formula used before [13]: Restriction = (100 − (% transformed B cells in co-culture/% transformed B cells without NK) ×100).

We found that CD56^bright^NKG2A^+^ NK cells inhibit EBV-induced B cell transformation greater than 6-fold more than their counterparts (*p* = 0.0005 for CD56^bright^NKG2A^−^ NK cells and *p* = 0.0002 for CD56^dim^ NK cells, respectively) (Figure 1C). In addition, CD56^bright^NKG2A^+^ readily produced IFN-γ upon IL-12 stimulation (10 ng/ml for 18 hours) (Figure 1D), another hallmark of the tonsillar anti-EBV NK subset identified by us previously [13].

Thus, CD56^bright^ and NKG2A expression sufficiently define phenotypically the potent anti-EBV NK subset, which is potent in IFN-γ production. We use these phenotype markers throughout this study to identify and functionally characterize this NK cell subset further.

### Tonsillar naïve B cells and centrocytes are more susceptible to EBV infection than memory B cells

**Figure 2:**
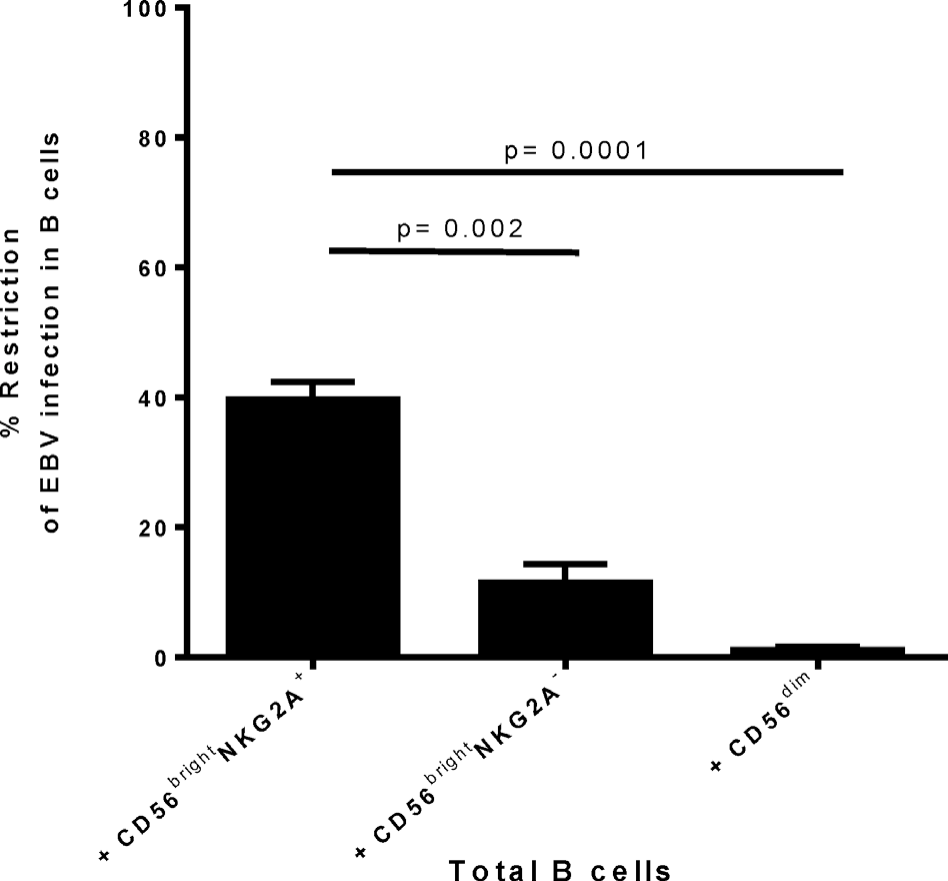
Differences in restriction of early EBV infection in B cells by autologous CD56brightNKG2A+ NK cells compared to other tonsillar NK subsets Restriction of transformation assay was adapted to analyze restriction of early EBV infection after 72 h post infection, i.e. before transformation is induced. EBV GFP tagged virus was used. Frequency of EBV positive cells was determined by frequency of GFP positivity. Restriction of EBV infection was calculated by formula: Restriction = (100 − (% infected cells in co-culture/ % infected cells without NK) ×100). Data represent three independent experiments, with a total of 3 donors. All error bars represent SEM. *p* values depicted were calculated with two-tailed student's *t* test.

Successively, we determined if the superior restriction capacity of CD56^bright^NKG2A^+^ NK cells compared to other NK cell subpopulations is detectable already early after infection with EBV. To this end, we adapted the B cell transformation assay and co-cultured flow-sorted autologous B cells with the distinct tonsillar NK cell subsets for 72 hours. As we used the recombinant GFP-EBV virus [16], we could determine the extent of B cell infection by flow cytometry measuring the frequency of GFP expressing B cells. By flow-sorting the B cell subsets before infection we ensured that any potential subset defining marker/ receptor changes upon culture and/or infection would not influence the analysis of the subsets susceptibility. Thus, we determined the true susceptibility of distinct B cell differentiation stages *in vitro*. We readily detected the potent CD56^bright^NKG2A^+^ NK cell-mediated restriction of EBV 3 days (72hours) after infection (Figure 2). CD56^bright^NKG2A^+^ NK cell restricted EBV-infected B cells greater than 3-fold more than CD56^bright^NKG2A^−^ NK cells (*p* = 0.002) and greater than 30-fold more than CD56^dim^ NK cells (*p* = 0.0001), respectively (Figure 2).

**Figure 3:**
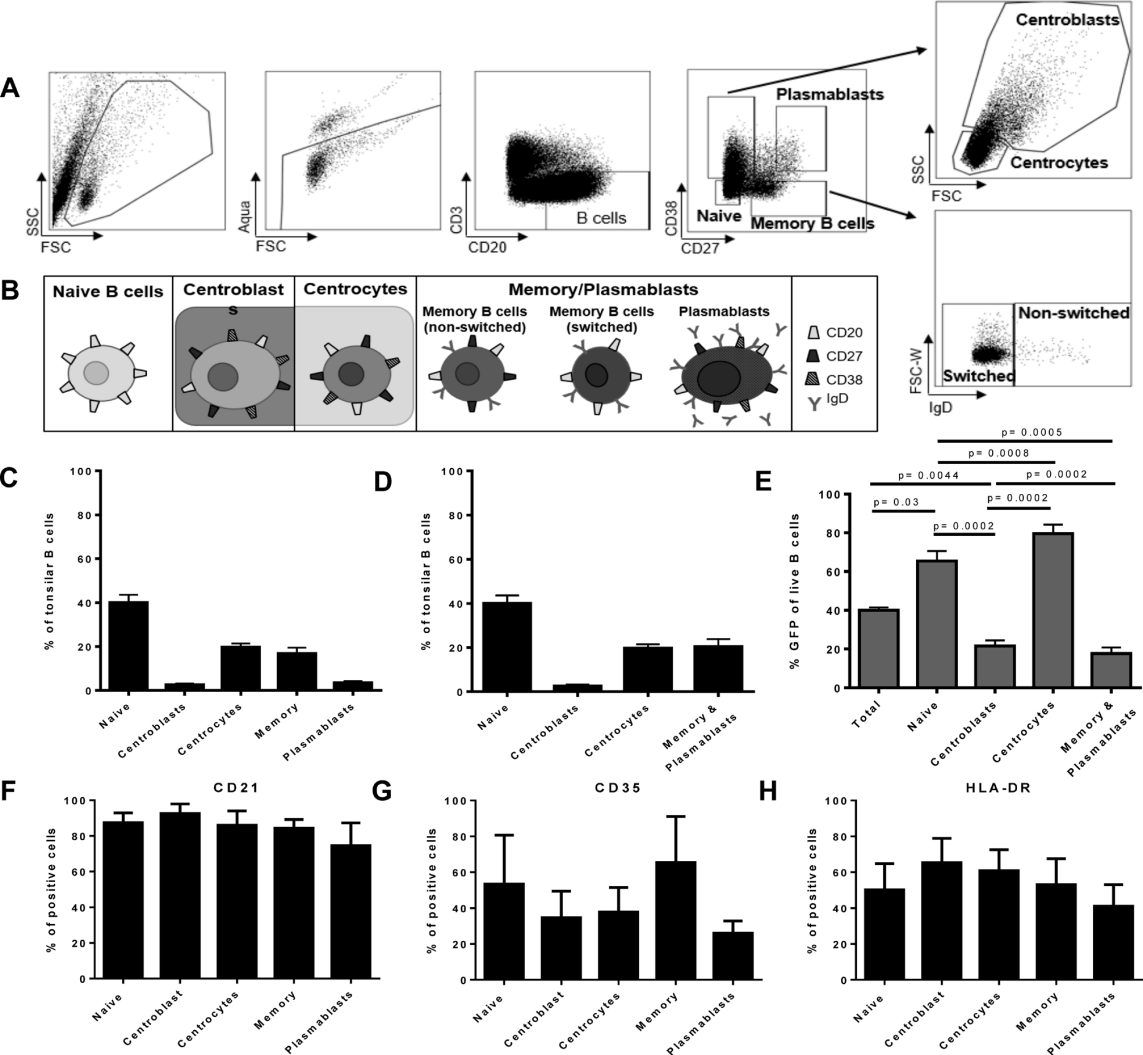
Flow-sorted human germinal center and pre-germinal center B cell subsets show higher susceptibility to EBV than post-germinal center subsets (**A**) Human tonsillar B cell subset gating; (**B**) Definition of human tonsillar B cell subsets for sorting experiments, higher maturation stages were grouped together as depicted; (**C**) Frequencies of all major tonsillar B cell subsets (4 donors, 3 experiments); (**D**) Frequency of the four investigated sorting subsets in tonsils (4 donors, 3 experiments); (**E**) Susceptibility of defined sorted tonsillar B cell subsets to EBV infection (72 h) (8 donors, 6 experiments). Frequency of EBV entry receptors CD21 positive (**F**), CD35 positive (**G**), or HLA-DR positive (**H**) cells in tonsillar B cell subsets (each 4 donors, 3 experiments); Frequency of all error bars represent SEM; p values depicted were calculated with two-tailed student's *t* test.

EBV-associated cancers are derived of germinal center B cells, i.e. centrocytes [5, 17, 18]. Which tonsillar B cell subsets are most susceptible to EBV infection *in vitro* remains debated and seems to depend on the experimental approach [5, 19–22]. Consequently, to analyze the potency of CD56^bright^NKG2A^+^ NK cells towards tonsillar B cell differentiation stages as intended, we first determined the susceptibility to EBV infection of the distinct tonsillar B cell differentiation stages *in vitro* without NK cells present, analyzing sorted naïve B cells, centroblasts, centrocytes and memory/plasmablasts B cells, respectively (Gating/Sorting strategy: Figure 3A). For all sorts, we grouped together memory B cells and plasmablasts (memory/plasmablasts, defined in Figure 3B). The distinct B cell differentiation subsets were sorted from EBV-naïve donors’ tonsillar mononuclear cells (TMCs), inoculated with recombinant GFP expressing-EBV [16], and the frequency of EBV-infected B cells was determined by flow cytometry after 72 hours. Frequencies of all B cell subsets (Figure 3C), as well as for the sorted subsets (Figure 3D) were recorded. After 72 hours of EBV inoculation, we found that naïve B cells and centrocytes are most susceptible to EBV infection of all analyzed differentiation stages (Figure 3E). Subsequently, we analyzed the increased susceptibility of naïve B cells and centrocytes towards EBV infection at very early time-points from 18 hours until 72 hours after infection. We detected the superior infection of naïve B-cells and centrocytes compared to centroblasts and memory/plasmablasts already at 18 hours after infection, and the fold-changes increased over time (data not shown).

To determine if these differences were due to differential expressions of known EBV entry receptors, we analyzed expression of CD21 (Figure 3F), CD35 (Figure 3G) and HLA-DR (Figure 3H) on the B cell subsets. We did not detect differential expression by the subsets in line with the susceptibility differences (Figure 3F, 3G, 3H).

Hence, we show here that tonsillar naïve B cells and centrocytes, which are undergoing or will undergo the germinal center reaction, are more susceptible to EBV infection as compared to centroblasts and higher differentiation stages of B lineage cells, and this is not dependent on differential expression of known entry receptors.

### Tonsillar naïve B cells and centrocytes are more susceptible to CD56^bright^ NKG2A^+^ NK cell-mediated restriction of EBV infection than higher differentiated B-cells

**Figure 4:**
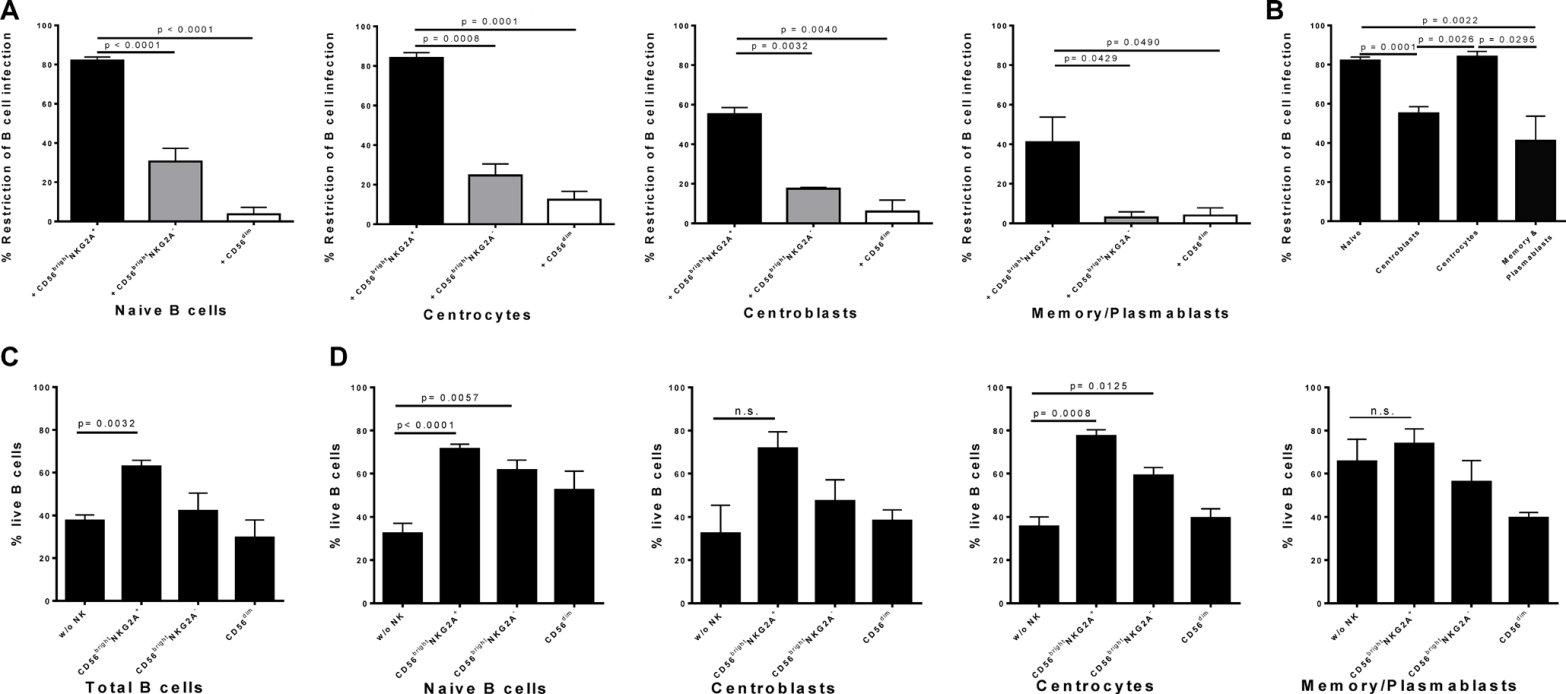
CD56brightNKG2A+ NK cells restrict EBV most potently in germinal center and pre-germinal center tonsillar B cell subsets (**A**) CD56^bright^NKG2A^+^ mediated restriction of EBV-infected B cell subsets compared to effects of CD56^bright^NKG2A^−^ and CD56^dim^ NK cells (8 donors, 6 experiments); (**B**) CD56^bright^NKG2A^+^-mediated restriction in EBV-infected B cell subsets after 72 h (8 donors, 6 experiments); Differences in survival in cultures without NK cells present and co-cultures of total B cells (6 donors, 4 experiments) (**C**) and of sorted B cell subsets (6 donors, 4 experiments) (**D**), measured by frequency of Live/Dead cell staining negative population at 72 h. All error bars represent SEM. *p* values depicted were calculated with two-tailed student's *t* test. n.s.- not significant.

**Figure 5:**
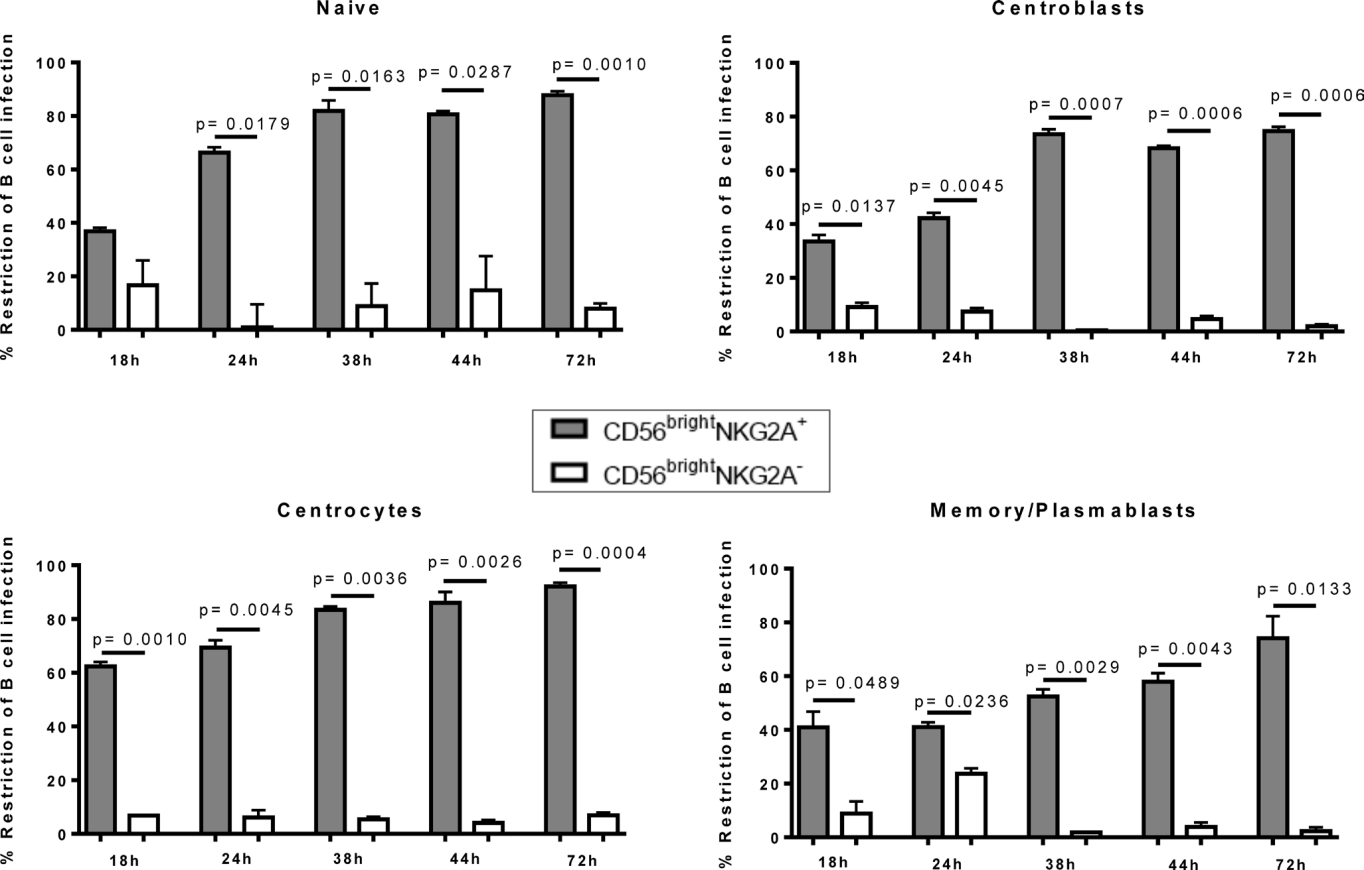
Early restriction of EBV by human CD56brightNKG2A+ NK cells over time Potency of CD56^bright^NKG2A^+^ mediated EBV restriction at different time points in flow sorted B cell differentiation stages compared to effects of CD56^bright^NKG2A^−^ NK cells. Data represent four independent experiments, with a total of 8 donors. All error bars represent SEM. *p* values depicted were calculated with two-tailed student's *t* test.

To determine if distinct pre-, post- or germinal center B-cell subsets are restricted at similar, or different levels by CD56^bright^ NKG2A^+^ NK cells, we next analyzed for the first time the potency to restrict EBV infection in distinct differentiation stages of B cells after 72 hours. CD56^bright^ NKG2A^+^ NK cells were more efficient in inhibiting the outgrowth of EBV-infected B cell subsets as compared to CD56^bright^NKG2A^−^ NK cells, and CD56^dim^ NK cells for each subset investigated (Figure 4A). CD56^bright^NKG2A^+^ NK cell-mediated restriction of EBV infection was most efficient for naïve B cells and centrocytes compared to centroblasts and memory B cells (Figure 4B), while memory/plasmablasts B cells were least susceptible. In the same assay, B cells showed significantly higher frequencies of live cells in co-cultures of total B cells with CD56^bright^NKG2A^+^ NK cells compared to cultures without NK cells, whereas the presence of CD56^bright^NKG2A^−^ or CD56 dim NK cells did not alter the frequency of live B cells (Figure 4C). When B cell differentiation stages were sorted before infection both co-cultures with CD56^bright^NKG2A^+^ and CD56^bright^NKG2A^−^ show higher frequencies of live B cells than cultures without NK cells, but only in naïve B cells and centrocytes. Furthermore, we observed potent inhibition of EBV-infected B cell outgrowth by CD56^bright^ NKG2A^+^ NK cells already 18 hours after EBV infection and it increased over time for all B cell subsets investigated (Figure 5).

In conclusion, we show here for the first time that CD56^bright^ NKG2A^+^ NK cells efficiently inhibit outgrowth of all tonsillar EBV-infected B cell differentiation subsets., being superior in their The restriction of EBV-infected naïve B cells and centrocytes was superior, compared to higher differentiation stages of tonsillar B cells.

### CD56^bright^ NKG2A^+^ NK cells restrict outgrowth of tonsillar EBV-infected B cells through IFN-γ and NKp44

**Figure 6:**
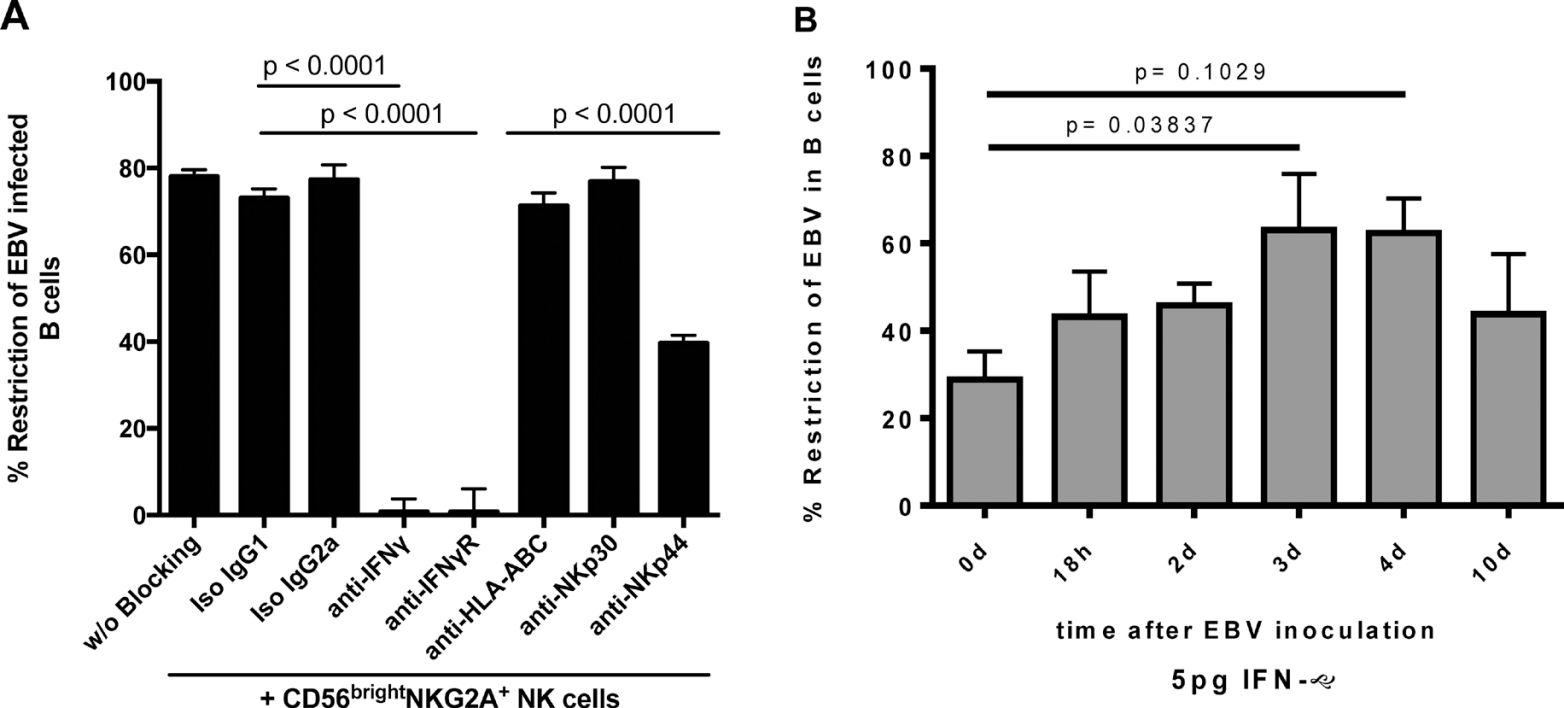
CD56brightNKG2A+-mediated restriction of EBV depends strongly on IFN-γ and partially on NKp44 (**A**) Blocking of NK cell receptors and IFN-γ or IFN-γ receptor (72 h) (8 donors, 4 experiments); (**B**) Low dose exogenous IFN-γ assay to define time points when EBV restriction is potent (6 donors, 4 experiments). All error bars represent SEM; *p* values depicted were calculated with two-tailed student's *t* test.

To determine the mechanism by which CD56^bright^ NKG2A^+^ NK cells inhibit outgrowth of tonsillar EBV-infected B cells, we inhibited activating natural cytotoxicity receptors with established [23–28] blocking antibodies specific for NKp30, and NKp44, interaction with MHC class I-recognizing inhibitory receptors with an established [29–31] blocking antibody specific for HLA-A, -B, and -C molecules and IFN-γ-mediated effects with antibodies specific for the IFN-γ and its receptor (Figure 6A). Restriction of EBV-infected B cells was determined 72 hours after infection as described above. Both blocking either IFN-γ or IFN-γ receptor (IFN-γR) abrogated the CD56^bright^NKG2A^+^ NK cell-mediated restriction of EBV. NKp44-specific antibody blockade inhibited EBV restriction, whereas blockade with NKp30 and HLA-ABC-specific antibodies did not show any significant effects. Next, we added low concentrations (5 pg/ml) of established [23, 24] exogenous IFN-γ to tonsillar B cells at various time points after EBV inoculation and monitored its efficacy to inhibit B cell transformation. IFN-γ alone potently induced restriction of EBV-infected B cell outgrowth, being most potent at days 3 and 4 after infection (Figure 6B). We included a dosage curve for IFN-g in the Supplementary Figure S2. Of note, due to IFN-g toxicity in higher concentrations, overall survival decreases markedly in cultures with higher concentrations and thus restriction in higher doses might be underestimated.

Taken together, CD56^bright^NKG2A^+^ NK cells restrict the outgrowth of tonsillar EBV-infected B cells through IFN-γ release and the natural cytotoxicity receptor NKp44 partially contributes to this effect and IFN-γ can restrict EBV even after infection and/or transformation is established.

## DISCUSSION

In this study we analyzed the innate NK cell response towards EBV infection in tonsillar B cells i.e. at the site of the first contact of EBV with B cells upon *in vivo* infection. We identified tonsillar CD56^bright^NKG2A^+^ NK cells as the main effector NK cell subset in restricting outgrowth of EBV-infected tonsillar B cells at the viral portal of entry. CD56^bright^NKG2A^+^ NK cells exhibited restriction as early as 18 hours after infection, and this was most efficient against EBV-infected naïve B cells and centrocytes, i.e. those B cell differentiation subsets we determined to be most susceptible to EBV infection. This process was predominantly mediated through IFN-γ release, and partially by the activating receptor NKp44. Our results may have implications for the use of NK cell subsets in novel treatments against EBV-associated B-cell malignancies.

We show here for the first time that CD56^bright^NKG2A^+^ NK cells from palatine tonsils are the most efficient tonsillar NK cell subset in impairing the outgrowth of EBV-infected B cells. In the past, only T cells were believed to play the crucial role in controlling established EBV infection [2]. Recent studies, however, provided increasing evidence that NK cells are contributing to the immune control [12, 13]. In particular, it was shown that NKG2A^+^ NK subsets accumulate *in vivo* in humans with infectious mononucleosis [15] and in experimental animal models such as humanized mice [14]. CD56^bright^NKG2A^+^ NK cells are precursors of CD56^dim^NKG2A^+^ cells, the NKG2A^+^ NK subset that accumulates in peripheral blood of patients with acute infectious mononucleosis [15]. This may suggest that the tonsillar CD56^bright^NKG2A^+^ NK cells differentiate into CD56^dim^NKG2A^+^ cells upon encounter of EBV. During infection with the human cytomegalovirus (CMV) [32], by contrast, NKG2C^+^ NK cells are expanded over long time periods in the peripheral blood, which in parallel acquired CD57 [33]. NKG2C^+^ cell expansions have also been described in infections with Hantavirus [34], Chikungunya virus [35], HIV [36, 37], and Kaposi Sarkoma in AIDS patients [38]. All of these expanded NK cell subsets represent high maturation stages and include classical killer cells and potent mediators of antibody-dependent cytotoxicity (ADCC) [39]. These high maturation stages of NK cells have been found to be potent effectors of the NK cell-mediated regulatory “rheostat” effects, protecting the viral niche by tuning down CD4 T cells, which regulate antiviral CD8 T cells [40]. Thus, NKG2A^+^ NK cell accumulation appears to be fairly specific for EBV. Our finding that tonsillar CD56^bright^NKG2A^+^ NK cells are the most efficient NK cell subset in restricting EBV strongly suggests that the CD56^dim^NKG2A^+^ NK cells observed in peripheral blood from patients with acute infectious mononucleosis may derive from their precursors in the portal of entry of EBV upon primary infection with this virus.

Our observation that tonsillar NKG2A+ NK cells predominantly elicit the restriction of EBV might be very remarkable in the light that subsequent to IM the risk to develop EBV-positive Hodgkin's lymphoma is 20-fold increased [4]. Thus, failure to efficiently control the acute primary EBV infection mirrored by an exuberant cytotoxic T cell response as in IM seems to predispose to EBV-associated B-cell cancer. Indeed, children younger than 5 years of age, who very rarely develop IM and EBV-associated B-cell cancer [2], have higher numbers of NKG2A^+^ NK cells in peripheral blood [41, 42]. In line with the strongly reduced frequencies of immature NKG2A^+^ NK cells found in adults [41, 42], we previously have shown that CD56^bright^NKG2A^+^CD94^+^CD54^+^CD62L^−^ NK cells are much less frequent in the peripheral blood of adults than of children [13]. Here we show that NKG2A^+^ NK cells in tonsils are the distinct NK cell subset exhibiting superior control of EBV-infected B cells. Most notably, we show that this subset is most potent against EBV-infected naïve B-cells which need to undergo the germinal center reaction and against centrocytes, the main cellular origin of EBV-associated B cell tumors [5]. Taken together, if EBV infection is deferred to later in life, the numbers of CD56^bright^ NK cells of potent anti-EBV CD56^bright^NKG2A^+^ NK cells present in tonsils to immediately contain the infection of the B cells might be too low so that the exuberant IM reaction is needed to control primary EBV infection. The transiently uncontrolled EBV infection of the naïve and germinal center B cells might predispose to increased somatic hypermutation and subsequently enhanced risk for “seeding” and developing cancer. We hypothesize that if CD56^bright^NKG2A^+^ NK cell mediated control is lacking or malfunctioning, cancer might develop driven by the strong transforming capacities of EBV at primary infection. This would be a potential explanation for the increased risk of EBV-associated cancers after primary EBV infection also whilst on iatrogenic immunosuppression, such as PTLD.

Equally important, our findings unprecedentedly demonstrate that CD56^bright^NKG2A^+^ NK cells restrict outgrowth of EBV-infected B cells dependently of IFN-γ, partially dependently of NKp44, and independently of NKp30 and HLA ABC. It was shown before, that NK cells mediate restriction of EBV-induced B cell transformation largely, but not completely, via IFN-γ [12, 13]. NK cell effector functions are controlled by germline-encoded activating and inhibitory receptors. If more activating signals are received due to high expression of activating NK cell receptor ligands or loss of inhibitory NK cell receptor ligands, NK cells are unleashed. This prompted us to explore whether such activating signals are involved in the outgrowth restriction. Therefore, we additionally functionally explored the restriction role of the activating receptors NKp44, NKp30, and NKG2D [43]. Indeed, we found reduction of restricting activity only for NKp44 (Figure 6A). Notably, only few blood NK cells express NKp44, whereas tonsillar NK cells express it more abundantly (Supplementary Figure S1). Thus, our data are in line with former studies, but document for the first time IFN-γ as the main mediator and of NKp44 as partial mediator early in infection resulting in restriction of outgrowth EBV-infected B cells.

Notably, we show that exogenous IFN-γ potently restricts EBV-induced transformation, even at low concentrations and even if added up to 10 days after *in vitro* infection (latest time point analyzed) to the B cell cultures. In 1985, IFN-γ was found to be potent against EBV up to 3–4 days after infection, whereas IFN-α and -β were found to be antivirally effective only within the first 24 hours after infection [44]. In contrast to our finding, in their assay of B cell outgrowth Lotz et al. more than 20 years ago found no relevant restriction following treatment more than 4 days after infection. They stated that their finding might be dose and assay dependent. Consequently, we suggest that the findings at variance might be explained by methodological differences and their sensitivities. Of further note, IFN-γ reached in our study the peak of restriction capacity with addition 3–4 days after infection. In this study, we added NK cells at the time of inoculation in physiological numbers and the restriction was mainly IFN-γ dependent. Thus, we might likely underestimate the anti-EBV strength of CD56^bright^NKG2A^+^ NK cells in our *in vitro* experimental set-up as these regulators might still be recruited to the sight of infection in higher numbers *in vivo* after initial infection.

One limitation of the present study is that we have not formally shown that NKG2A from tonsillar NK cells binds to HLA-E. Nevertheless, there is ample experimental evidence regarding the specificity of NKG2A and HLA-E binding [45–48]. The inhibitory CD94/NKG2A receptor belongs to the C-type lectin family of proteins [45]. It binds to the non-classical MHC I molecule HLA-E [46] and results in an inhibitory signal. Patients with acute hepatitis B virus infection were found to have increased numbers of NKG2A^+^ NK cells in their peripheral blood and blocking CD94/NKG2A interaction with HLA-E was found to promote clearance of hepatitis B virus very efficiently in mice [47]. In some cases, e.g., in some cancer cells, HLA-E is upregulated to evade attack of the anti-cancer NK cells, and very recently a “humanized” blocking antibody has been shown to potently unleash their potential against human leukemic cells and EBV cell lines in engrafted mice and rescue them from disease progression [48]. In line with this, Monalizumab by Innate Pharma/AstraZeneca (London) is an anti-NKG2A antibody infusion currently in phase 1 and 2 trials against head & neck cancers. This antibody therapy blocks the inhibition signal of the NKG2A receptor, and thereby unleashes those cells towards cancer cells, which can express high levels of the NKG2A ligand HLA-E. Our data indicate that NKG2A blockade could be harnessed against EBV positive B cell cancers.

In conclusion, we identified human tonsillar CD56^bright^NKG2A^+^ NK cells to restrict outgrowth of autologous EBV-infected tonsillar B cells, preferentially targeting germinal center or pre-germinal center (naïve) B cell subsets. Germinal center B cells are strongly associated with EBV-associated cancers [5, 17, 18]. Therefore, unleashing and harnessing CD56^bright^NKG2A^+^ NK cell function by e.g. either by blocking HLA-E on tumor cells, or by blocking NKG2A^+^ on NK cells or administering cytokines such as IL-15 which expand these NK cell subsets in the patients, might have a strong therapeutic merit in limiting and fighting EBV-associated tumor formation.

## MATERIALS AND METHODS

### Ethical approval for human samples

All studies were conducted in accordance with the guidelines of the World Medical Association's Declaration of Helsinki. All studies were reviewed and approved by the institutional review board protocol KEK-StV-Nr. 19/08 through the cantonal ethical committee of Zurich, Switzerland. After informed consent of the donors legal guardians blood and tonsil samples were received for study purposes. The Zürcher Blutspendedienst SRK, Switzerland, who had also previously approved all studies, provided blood concentrates.

### Reagents

The following anti-human Abs were used in this study: 7-AAD PerCP, CD8 PE, CD16 APC-Cy7, CD21 APC, CD38 PE, CD56 PE-Cy7, DNAM-1 FITC, GM-CSF PerCP/Cy5.5, IFN-γ APC, Ki 67 FITC (all BD Biosciences); CD3 Pacific Blue (Life Technologies); CD4 APC, CD19 PE, (eBioscience); CD20 PE-Cy5, CD27 APC-Cy7, IgD PE-Cy7, NKp44 APC (BioLegend); CD158a, h PE-Cy5.5, CD158b1/b2, j PE-Cy5.5, CD158a, h APC, CD158b1/b2, j APC, CD159a (NKG2A) PE, CD159a (NKG2A) APC (Beckmann Coulter); hIL-12 Rß PerCP (R&D Systems). Live cells were distinguished using the LIVE/DEAD Fixable Aqua Dead Cell Stain Kit (Life Technologies). Following endotoxin free recombinant human (rh) cytokines were used: rhIL-12 (R&D Systems), rhIL-2 (PeproTech), and rhIL-15 (Sigma).

### EBV preparation, cell culture and serostatus of donors

Rekombinant GFP-tagged EBV was produced in the B95-8 bacmid use [16] carrying 293-HEK cells in 10 cm dishes by standard 4-h Metafectene Pro (Lucerna) transfection according to the manufacturer's protocol. Briefly, after selction by 0.1% Hygromycin in the medium to 70% confluency, plasmids (p509 carrying BZLF-1 [49]; and p2670 carrying BALF-4, gp110 [50]) were preincubated 20 min at room temperature with Metafectene and then added to the cell dishes and incubated 4 h in antibiotic-free medium. Then, RPMI 1640 + 10% FCS medium was replaced with gentamicin-containing RPMI 1640 + 10% FCS medium. After 3 days supernatant was harvested and filtered through 0.45-μm filters, and EBV was further purified and concentrated by ultracentrifugation at 30,000g for 2 h at 4°C, resuspended carefully in RPMI 1640, and titered on Raji cells [51] as described previously [13]. Titers were calculated in Raji infectious units (RIU) and Titers of 0.5RIU were used for all experiments.

All cells and cell lines were cultured at 37°C, 5% CO_2_ if not noted otherwise.

For all tonsil samples serology for EBV was determined with the ImmunoDot Mono G Kit (GenBio) on blood plasma.

### NK and B cell purification and culture

To obtain Peripheral Blood Mononuclear Cells (PBMCs), blood samples from under-age tonsillectomy patients of the University Children's Hospital Zurich or from adult donors by the Zürcher Blutspendedienst SRK were received within 18 hours after blood withdrawal. Single cell suspension from tonsils were obtained as described before [13]. TMCs and PBMCs were then isolated by Ficoll-Paque PREMIUM (GE Healthcare) gradient centrifugation and cryopreserved.

After thawing TMCs or PBMCs cells were washed in PBS. Cell suspension was filtered through a 70 μm nylon cell strainer to remove debris and dead cells. The various cell types were purified from PBMC or TMC samples via fluorescence-activated cell sorting (FACS) or magnetic cell separation (MACS) for separating B and NK cells and their subsets as indicated in the figure legends.

For some experiments, total B cells were purified by positive selection kit human CD20 MicroBeads on the QuadroMACS^™^ Separator and corresponding MACS MultiStand (Miltenyi biotec), as described in the manufacturer's protocol. The CD20 negative fraction was consecutively used for isolation of total NK cells, which were extracted via the human NK Cell Isolation kit through negative selection according to the manufacturer's protocol (Miltenyi biotec).

For all other experiments, cells and subsets were isolated via flow sorting on the Aria III (BD Biosciences). Briefly, cells were suspended in 2% hi-FBS PBS before sorting into B cells (CD3^−^ CD20^+^) and NK cells (CD3^−^ CD56^+^) on the BD FACSAria III through an 85 μm nozzle for 4-way purity. In a second step, NK cells were furthermore sorted into CD56^bright^NKG2A^+^ NK cells CD56^bright^NKG2A^−^ and CD56^dim^ NK cells, and B cells were into naïve B cells (CD3^−^CD20^+^CD38^−^CD27^−^), centrocytes (CD3^−^CD20^+^CD38^+^CD27^+/−^ and small size), centroblasts (CD3^−^CD20^+^CD38^+^ CD27^+/−^ and bigger size) and memory/ plasmablasts (combining non-switched, switched memory B cells and plasmablasts). Detailed gating strategies applied for B and NK cell sorting are represented in Figures 1A and 1B and 2A. Throughout this study, purity of cell isolates was > 97 % for all experiments.

### B cell transformation/infection assays

The transformation assay was performed as described previously [13]. In NK co-cultures 1’500 sorted NK cells were added to 100’000 B cells to mimic physiologic proportions in tonsils (ET ratio 1:40) as previously established [13]. Non-virus inoculated samples as well as NK-cell free cultures served as controls for infection as well as restriction rates. The infection assay was adapted from the transformation assay [13]. Cells were kept in culture for 18, 24, 38, 44 or 72 h. GFP expression was analysed via flow cytometry (FACSCanto II, BD). Restriction of infection was calculated with the adapted formula described previously for restriction of transformation [13], i.e. Percentage of restriction of B cell infection = (1 - total GFP + B cells of sample with NK cells/ total GFP + B cells of sample without NK cells) × 100. For blocking experiments, applicable cells were treated as described before [52]. Briefly, cells were preincubated for 1 hour with 10 μg/ml neutralizing antibodies: α-CD337 (NKp30), α-HLA-A, -B, -C, α-IFN-γ or α-CD336 (NKp44) (all BioLegend) or with isotype controls α-IgG1Κ and α-IgG2a (eBioscience). Cells were (co-)cultured for 72 h and infection status of B cells as well as B cell survival rates were determined by flow cytometry.

For the IFN-γ dose-response assay, flow sorted tonsil-derived B cells of 5 donors were plated à 1 × 10^5^ cells per well in a flat-bottom 96-well plate. After inoculation with 0.5 MOI of recombinant EBV cells were cultured for 12 d, 0 h, 18 h, 2 d, 3 d, 4 d or 10 d after inoculation different amounts of recombinant human IFN-γ (rhIFN-γ, BioLegend) were added. To prevent starvation, cells were fed at days 4 and 8 with 50 μL R10.

### Flow cytometry, data analysis and statistics

General gating strategy for live cells: lymphocyte population in forward/sideward scatter (FSC/SSC), live (Aqua-) cells. All flow cytometry data analysis was performed with FlowJo Version ×.0.7. For statistical evaluation and representation of data we used GraphPad Prism software Version 10. Unpaired *t*-test, regular ANOVA Tukey-corrected for multiple comparisons were applied as indicated in the figure legends, and *p*-value ≤ 0.05 was defined as statistically significant. Error bars in the graphs and figures represent the standard error of the mean (SEM).

In addition, the authors would like to thank the flow cytometry facility of the University of Zurich for maintenance of the BD Aria III and acknowledge that all cell sorting was performed with their equipment.

## SUPPLEMENTARY MATERIALS FIGURES AND TABLES


