# Supplementary material for: Tonsillar CD56brightNKG2A+ NK cells restrict primary Epstein-Barr virus infection in B cells via IFN-γ
Source: Oncotarget. 2016 Dec 20;8(4):6130–41. doi: 10.18632/oncotarget.14045 (PMC5351618; doi:10.18632/oncotarget.14045)
Supplement: Supplementary file 1 [file oncotarget-08-6130-s001.pdf]

Tonsillar CD56^{bright}NKG2A^{+} NK cells restrict primary Epstein-Barr virus infection in B cells via IFN-γ

Supplementary Materials

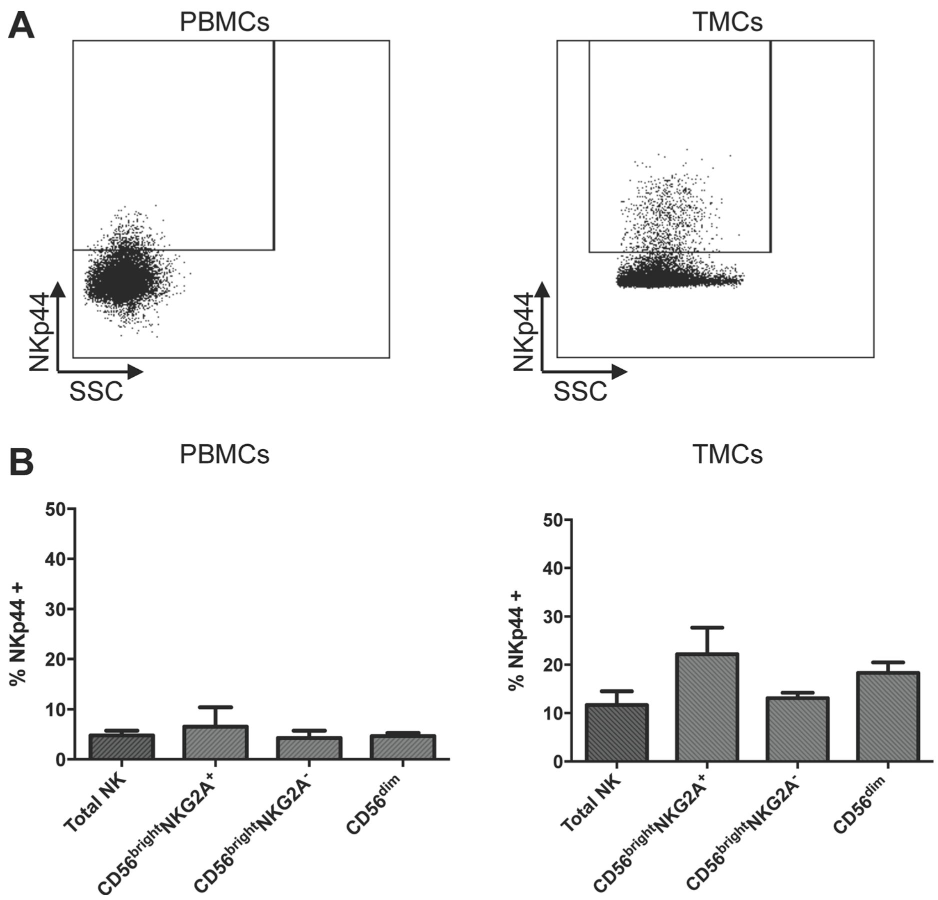

Supplementary Figure S1: NKp44 expression is limited to few NK cells in the blood, and is expressed more abundantly in tonsilar NK. PBMCs and TMCs were analyzed *ex vivo* for their frequency of NKp44 expressing cells. NK subsets were compared. (**A**) Dot blots of total NK cells from PBMCs and TMCs depicting NKp44 expression (*ex vivo*), representative donors are shown; (**B**) Data of frequency of NKp44 positive cells in NK subsets and total NK is summarized from 5 TMC and 8 PBMC donors in 3 independent experiments; Bar graphs depict the mean ± SEM; two-tailed student's *t* test was not significant for all comparisons ($p < 0.05$).

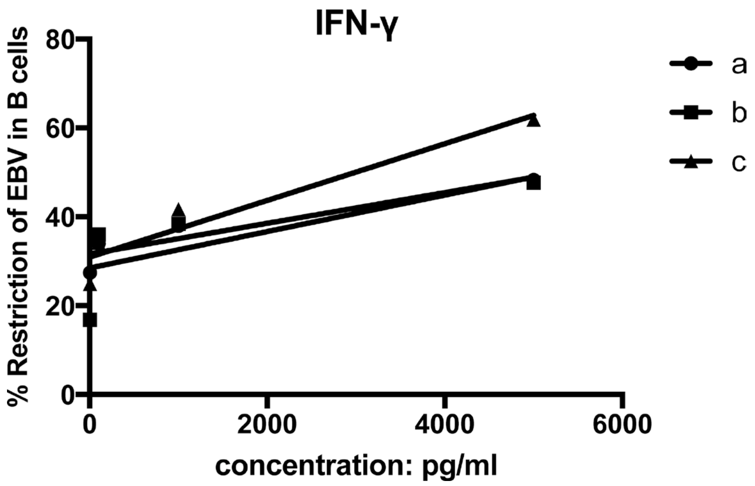

Supplementary Figure S2: Dose-response curve of exogenous IFN-γ. Purified B cells were infected with EBV and exogenous IFN-g was added at the same time (d0). After 12 days, EBV transformation of B cells was determined and restriction of EBV in B cells was calculated relative to cultures with no exogenous IFN-g added. Three experiments with a total of three donors each in triplicates are shown.
